# Is perceived safety and threat after workplace terrorism linked to employee sick-leave? A registry-based longitudinal study of governmental employees in Norway

**DOI:** 10.1080/20008198.2020.1785249

**Published:** 2020-08-11

**Authors:** Alexander Nissen, Mona Berthelsen, Maria Teresa Grønning Dale, Marianne Bang Hansen, Trond Heir

**Affiliations:** a Division for Forced Migration and Disaster Research, Norwegian Centre for Violence and Traumatic Stress Studies, Oslo, Norway; b Department of Psychology, University of Oslo, Oslo, Norway; c National Centre for Hearing Impairment and Mental Health, Clinic for Mental Health and Addiction, Oslo University Hospital, Oslo, Norway; d Institute of Clinical Medicine, University of Oslo, Oslo, Norway

**Keywords:** perceived safety, perceived threat, sick-leave, health, terrorism, workplace, seguridad percibida, amenaza percibida, ausencia por enfermedad, saludterrorismo, lugar de trabajo, 感知安全, 感知威胁, 请假, 健康, 恐怖主义, 工作场所, •Terrorism enhances fears and undermines perceived safety in both directly and indirectly exposed individuals, and this may negatively impact health.•The workplace is often targeted in terrorist attacks and could play a role in mitigating consequences.•The study explored employee perceived threat/safety and subsequent sick-leave after a workplace terrorist attack.•Results suggest that employees with high perceived safety have less sick-leave, even after adjusting for terrorism exposure and symptom-based PTSD.

## Abstract

**Background:**

A large body of research has shown that terrorism enhances fears and undermines perceived safety in a high proportion of both directly exposed individuals and individuals without any form of direct exposure (i.e. no geographical proximity to an attack). Some studies have further suggested that fear of terrorism may adversely affect health in those without direct exposure and that this may constitute an important public health burden because of the number who are indirectly exposed. Limited studies have investigated threat and safety perception after workplace terrorism and the possible consequences for employee health.

**Objective:**

To explore whether perceived safety and threat in employees whose workplace was subjected to a terrorist attack are associated with subsequent sick-leave.

**Method:**

A longitudinal questionnaire survey on governmental employees’ perceived safety and threat at work one (T1) and two (T2) years after the 2011 terrorist attack on the Norwegian ministries was linked to registry data on doctor-certified sick-leave for two 9-month periods following T1 and T2 (N = 1703).

**Results:**

There was fairly strong evidence (0.004 < *p* < 0.034) that higher perceived safety was associated with a close to 30% reduction in sick-leave in fully adjusted models which included terror exposure and symptom-based PTSD. There was inconclusive evidence that lower perceived threat was associated with reduced sick-leave in the full models.

**Conclusions:**

Reduced perceived safety in employees following workplace terrorism may have adverse health consequences of public health significance given how prevalent this perception seems to be. The study supports that post-terrorism response plans should include strategies on how to address the potentially large number of individuals suffering ill health after terror even if they were not directly exposed and do not meet criteria for PTSD.

## Abstract (es)

**Antecedentes**: Una gran cuerpo de investigación ha mostrado que el terrorismo incrementa el miedo y disminuye la percepción de seguridad en gran proporción tanto en individuos directamente expuestos como también en individuos sin ninguna forma de exposición directa (es decir, sin ninguna proximidad geográfica al ataque). Algunos estudios han sugerido que el miedo al terrorismo puede afectar en forma negativa la salud de aquellos sin exposición directa y que esto puede constituir una importante carga en la salud pública, debido al número de personas expuestas indirectamente. Un limitado número de estudios han investigado la percepción de amenaza y seguridad después de terrorismo en el lugar de trabajo y sus posibles consecuencias para la salud de los empleados.

**Objetivo**: Explorar si es que la seguridad y amenaza percibida en empleados en los cuales su lugar de trabajo fue sujeto a un acto terrorista están asociadas con permiso de ausencia por enfermedad.

**Método**: Se conectaron los resultados de un cuestionario longitudinal sobre la seguridad y amenaza percibida en empleados gubernamentales al primer (T1) y segundo (T2) año posterior al ataque terrorista de los ministerios noruegos del año 2011 con los datos registrados de permiso de ausencia por enfermedad certificados por un médico en dos períodos de nueve meses posteriores a T1 y T2 (N=1703)

**Resultados**: Se encontró evidencia bastante fuerte (0.004< *p* < 0.034) que mayor seguridad percibida se asoció con una reducción cercana al 30 por ciento en permiso por enfermedad en modelos completamente ajustados que incluían exposición al terror y TEPT basado en síntomas. Hubo evidencia poco concluyente de que menos percepción de la amenaza fue asociada con disminución en permiso por enfermedad en los modelos completos.

**Conclusiones**: La percepción reducida de seguridad en empleados posterior a terrorismo en el lugar de trabajo puede tener consecuencias de salud adversas de relevancia para la salud pública, debido a lo prevalente que es esta percepción. Este estudio apoya la tesis de que los planes de respuesta post-terrorista debiesen incluir estrategias sobre cómo lidiar con el número potencialmente grande de personas que sufren problemas de salud después del terror, incluso si no estuvieron expuestos directamente y no cumplen con los criterios para TEPT.

## Abstract (zh)

**背景**:大量研究表明, 恐怖主义在很大一部分直接暴露或无任何直接暴露形式 (即地理上与攻击不接近) 个体中, 增加了恐惧并破坏了人们的感知安全。一些研究进一步表明, 对恐怖主义的恐惧可能会对无直接暴露者的健康产生不利影响, 并且由于间接接触的人数众多, 这可能成为一个重要的公共健康负担。有限的研究考查了工作场所恐怖主义后的威胁和安全感及其对员工健康的可能影响。

**目的**:探讨工作场所遭受恐怖袭击的员工的感知安全和威胁是否与随后的病假有关。

方法:一项关于在2011年对挪威各部的恐怖袭击后一年 (T1) 和两年 (T2) 后政府雇员在工作中的感知安全和威胁的纵向调查问卷, 与T1和T2之后医生证明的9个月病假的注册数据相关联 (N = 1703) 。

**结果**:有相当强的证据 (0.004 <*p* <0.034) 表明, 在包括恐怖暴露和基于症状的PTSD在内的完全校正模型中, 较高的感知安全与病假减少近30％有关。没有结论性的证据表明, 在全模型中, 较低的感知威胁与病假减少相关。

**结论**:鉴于工作场所恐怖主义后员工的感知安全降低普遍存在, 降低的感知安全可能会对公共健康产生不良的健康后果。本研究支持恐怖主义后的应对计划应包括一些策略, 关于如何应对遭受恐怖袭击后健康状况不佳的潜在大量个体, 即使他们没有直接暴露在外, 也不符合PTSD的标准。

## 1. Background

On July 22nd, 2011, a massive car-bomb was detonated in downtown Oslo, Norway, by a politically motivated right-wing, Norwegian, extremist. The terrorist attack was directed against the Norwegian ministries and their employees. The attack killed eight people, wounded more than 200 others and caused extensive damage to infrastructure, forcing several ministries to relocate. Roughly 10% of the more than 3000 ministerial employees with offices in close proximity to the blast were at work when the bomb exploded (for more details on the attack, please see Nissen & Heir, 2016).

Terrorism heightens fears and undermines the feeling of safety, most markedly in individuals directly exposed to terrorist acts, but also in large groups of indirectly or even remotely exposed people (Boscarino, Adams, Figley, Galea, & Foa, 2006; Finseraas & Listhaug, 2013; Marshall et al., 2007; Rubin et al., 2007; Rubin, Brewin, Greenberg, Simpson, & Wessely, 2005; Schuster et al., 2001; Silver, Holman, McIntosh, Poulin, & Gil-Rivas, 2002). Limited research has explored threat and safety perception in employees whose workplace was targeted in a terrorist attack, even though the workplace is a common target for terrorism and could play a role in mitigation and recovery (Howie, 2007; North et al., 2010; Schouten, Callahan, & Bryant, 2004). Available evidence suggests these employees experience similar heightened fears and reduced safety at work (Grieger, Fullerton, & Ursano, 2003a; Grieger, Fullerton, Ursano, & Reeves, 2003b; Nissen, Birkeland Nielsen, Solberg, Bang Hansen, & Heir, 2015; Nissen, Hansen, Nielsen, Knardahl, & Heir, 2019). In fact, the experiences are no confined to employees whose workplace was directly affected by an attack (i.e. targeted or damaged), but have also been reported in employees without any form of direct exposure through their workplace (Howie, 2007; Mainiero & Gibson, 2003; North, Barney, & Pollio, 2015). Relatedly, exposure to other forms of workplace violence such as physical assaults, threats and verbal abuse has also been linked to the feeling of safety and threat in workers (Barling, Rogers, & Kelloway, 2001; Lanctôt & Guay, 2014).

Feeling safe has been considered important for human mental health and functioning for decades in psychology (e.g. Maslow, 1943). Within psychotraumatology and the cognitive model of posttraumatic stress disorder (PTSD), distorted threat and safety perception are thought to be key early features in the development of the disorder (Ehlers & Clark, 2000). In the context of intentional traumatic events at the workplace, including terrorism and other forms of violence, several studies support the cognitive PTSD model, showing that employees’ safety and threat perception are associated with both acute stress disorder (ASD) (Grieger et al., 2003b; Hansen & Elklit, 2011) and PTSD (Fullerton, Herberman Mash, Benevides, Morganstein, & Ursano, 2015; Grieger et al., 2003a; Nissen et al., 2015). Moreover, distortions in safety and threat perception became a more integrated part of the PTSD diagnosis with the latest version of the Diagnostic and Statistical Manual of Mental Disorders (DSM-5) through the D criterion: negative alterations in cognitions and mood (American Psychiatric Association, 2013). There is ample evidence documenting elevated rates of PTSD after terrorism, especially in directly exposed individuals, but also in individuals with indirect exposure (for an overview, please see Paz García-Vera, Sanz, & Gutiérrez, 2016). We therefore hypothesized that employees’ threat and safety perception would be associated with sick-leave through the close association with, or even causal role in PTSD.

Importantly, though, the health consequences linked to post-terrorism threat and safety perception may not be limited to those explained through direct exposure and PTSD. For example, long-lasting worry about terrorism could lead to sub-diagnostic mental distress which could subsequently impede health. As discussed above, there is quite strong evidence that a sizable part of the population experiences heightened fears and reduced safety after terrorism. Therefore, even if these perceptions confer a minimally increased risk for ill health, the aggregate population effect may be notable. Relevant to the present study, research has indicated that ongoing worry and fear of terrorism could negatively impact health by increasing low-grade inflammation and thereby the risk of cardiovascular disease (Melamed, Shirom, Toker, Berliner, & Shapira, 2004); by moderating the longer-term potential adverse cardiovascular effects of acute stress reactions to terrorism (Holman et al., 2008); and by mediating the negative effects of media exposure to terrorism on functional impairment due to physical and emotional health issues (Holman, Garfin, Lubens, & Silver, 2019). The study populations in these studies were either healthy young adults undergoing regular health screenings or representative national samples. The vast majority had no direct exposure to terrorism, though all were arguably indirectly exposed through the media.

Limited research has explored the health consequences of threat and safety perception in the setting of workplace terrorism aside from the aforementioned studies on ASD and PTSD. A few studies on workplace violence other than terrorism have linked perceived threat to more general measures of employee mental and somatic health (Barling et al., 2001; Rogers & Kelloway, 1997; Shiao et al., 2010). These studies, however, did not consider the potential mediating and/or confounding role of PTSD. An important aim of the present study, therefore, was to explore if perceived threat and safety may lead to sick-leave in the large group of ministerial employees who were not directly exposed to the attack and who do not meet symptom-criteria for PTSD. From an employer’s and public health point of view, this group may be important to study as even a small increase in the relative risk of ill health could amount to a large total burden on health due to the size of the group.

The majority of studies to date on how fear of terrorism may impact health in people with no direct exposure rely on self-report data. Using sickness absence as a measure of health may partly circumvent some of the methodological challenges associated with using self-report data (e.g. information bias). Several large studies have investigated the use of sickness absence as a global measure of health in working populations and found that it is a strong predictor of all cause mortality (Kivimäki et al., 2003; Vahtera, Pentti, & Kivimäki, 2004) as well as physical and mental health functioning (Mänty et al., 2017). Clear associations have also been found between sickness absence and self reported health (e.g. Marmot, Feeney, Shipley, North, & Syme, 1995). The evidence tends to be stronger if doctor-certified, longer-term, sickness absence is used (e.g. Kivimäki et al., 2003). Interestingly, one study found sub-clinical mental disorder to be associated with self-reported sickness absence (Rai, Skapinakis, Wiles, Lewis, & Araya, 2010), though this association was not found in another study where sickness absence was measured through sickness absence databases (Stansfeld, Fuhrer, & Head, 2011). By combining longitudinal self-report data with registry data on sick-leave in a large sample of employees whose workplace was targeted in a terrorist attack, the present study is well positioned to enhance current knowledge on the consequences of workplace terrorism. Specifically, the aim of the study is to explore whether employee threat and safety perception after workplace terrorism are associated with doctor-certified sickness absence.

## 2. Methods

### 2. 1. Design and participants

In the months following the terrorist attack, the Norwegian Centre for Violence and Traumatic Stress Studies and the National Institute of Occupational Health collaborated with the government occupational health services and the ministries themselves in planning and setting up a three-wave survey study involving all ministerial employees. The study focused on mental health and work environment factors in the aftermath of the attack, and included data on employee threat and safety perception. The study has permission to link survey data to individual-level data in national registry databases on sick-leave, health-care utilization and drug prescription for consenting participants. The present study was a small part of the larger study and investigated whether employee threat and safety perception after the attack was associated with subsequent sick-leave. The study combined data from the first two waves of the survey with registry data on doctor-certified sick-leave in the ensuing periods. The majority of the ministries conducted the first wave of the survey (T1) between April and July 2012, and the second wave (T2) between April and June 2013. Data from the third wave in the survey (T3) was not used in the present study because the ministries underwent major restructuring in the midst of the T3 data collection following the 2013 Norwegian parliamentary election. If the study had included the T3 survey, the risk would be that data on perceived threat and safety would refer to one work environment prior to restructuring and be used to predict sick-leave (outcome) in another work environment *after* restructuring (e.g. an employee who worked close to the epicentre of the blast prior to restructuring may have felt unsafe and had high sick-leave, though after the employee was relocated to a different ministry away from the epicentre, the employee may have felt safe and had low sick-leave).

Eligible participants included all employees in the Norwegian ministries at the time of the attack. Employees who left the ministerial system or changed ministry affiliation prior to the third survey wave (T3) and employees in ministries deviating from study procedures were excluded. Eligible participants received information about the study through emails, ministerial intranet sites and meetings in the fall of 2011 and winter of 2012, and were allowed to withdraw prior to study start or at any time during the study. Willing participants received a postal invitation letter (either via work or through their personal address) containing further information about the study and withdrawal procedures, a project specific ID-number and a login code to access the study’s online questionnaire. Participants were asked in the survey if they consented to survey data being linked to registry data on sick-leave, and had to answer affirmatively for sick-leave data to be obtained. The key to match project IDs with social security numbers was stored on a secure, offline server managed by an IT expert in accordance with ethical and data-security standards in Norway. The research team did not have access to the key nor the identity behind survey responses. Ethical approval was granted by the Regional Ethics Committee in Norway.

### 2. 2. Variables and measurements

Perceived safety and threat were measured through statement-questions scored on a 5-point scale ranging from 1 = disagree to 5 = agree. Perceived safety was measured with the statement: ‘I feel safe when I am at work’, which is part of the Safety Perception Scale constructed by Grieger et al. in connection to the 9/11 attacks in the US (Grieger et al., 2003a). The original scale contains two additional items: one concerning perceived safety at home and the other perceived safety in usual activities (overall Cronbach’s alpha for the three items in the original study = 0.78). These two items were omitted as the present study concerned safety perception at work only. Because there were few individuals in the lowest three answer categories (1–3), the three categories were combined in analyses. Perceived threat was measured with the statement: ‘I feel it is only a matter of time before my workplace is subjected to another terrorist attack’, taken in adapted form from Cox and Cheyne’s Safety climate Assessment Toolkit (Cox & Cheyne, 2000). The item was chosen because it showed the highest loading for personal appreciation of risk (standardized loading = 0.78). Because there were few individuals in the highest three answer categories (3–5), the three categories were combined in analyses.

**Figure 1.**
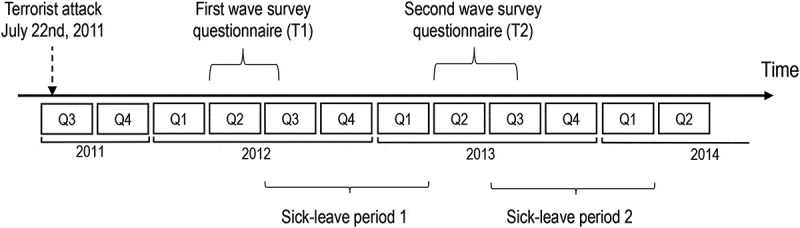
Timeline of study on sick leave in association with perceived safety and threat among employees in the Norwegian ministries after the 2011 terrorist attack in downtown Olso, Norway.

The main outcome variable, days of doctor-certified sickness absence, was based on registry data on employment from Statistics Norway and registry data on sick-leave from the Norwegian Labour and Welfare Administration. The former registry contains the total number of expected days of work, per quarter, for a person given the person’s contract(s) of employment. Public holidays, weekends and days of vacation are removed and not considered potential work days. A person working full-time has roughly 170 expected days of work for a 9-month period, which is the length used to measure sick-leave in the present study. The latter registry contains the number of days lost from work due to doctor-certified sickness absence, per quarter, for a person. The number takes into account if an employee works part-time and if sick-leave is graded. For example, if a person who works 80% (i.e. four days per week) gets two weeks of 50%, doctor-certified, sick-leave in a quarter, this person will have four registered days of sickness absence. Most ministries undertook the T1 and T2 surveys during quarters two of 2012 (T1) and 2013 (T2), and sick-leave was examined in the two 9-month periods following these survey points (see Figure 1). Sick-leave prior to the attack was included as a potential confounder and defined as sickness absence days from the first quarter of 2008 up until and including the second quarter of 2011 divided by the number of expected work days registered for the same period.

Symptom-based PTSD was measured through the Norwegian version of the 17-item PTSD Checklist (PCL). The PCL scale has been widely used in research on post-traumatic stress reactions and has shown good psychometric properties (Blanchard, Jones-Alexander, Buckley, & Forneris, 1996; Hem, Hussain, Wentzel-Larsen, & Heir, 2012; Weathers, Litz, Herman, Huska, & Keane, 1993). Earlier studies conclude somewhat differently in terms of how the scale is best used to identify likely PTSD and whether a single cut score or a combination of cut score and symptom cluster scores should be used (McDonald & Calhoun, 2010). We decided to use a score of ≥ 44 to define a PTSD case in the present study based on earlier research on civilian populations and because we wanted a cut score with fairly high sensitivity (Blanchard et al., 1996).

Terror exposure was approximated through employees’ whereabouts at the time of the attack. Participants could chose between the following answers: 1) in the government quarter in downtown Oslo; 2) in downtown Oslo, but not in the government quarter; 3) in Oslo, but not downtown; 4) In Norway but not in Oslo; and 5) abroad. The variable was dichotomized in analyses into ‘present’ (category one) versus ‘not-present in the government quarter’ (the other four categories).

The following demographic factors were included: age (continuous), gender and education. Education was split into the following three categories: <13 years (no years of study at university level); 13–16 years (some study at university level, but less than 4 years); and > 16 years (four or more years of study at university level).

### 2. 3. Statistical analyses

**Figure 2.**
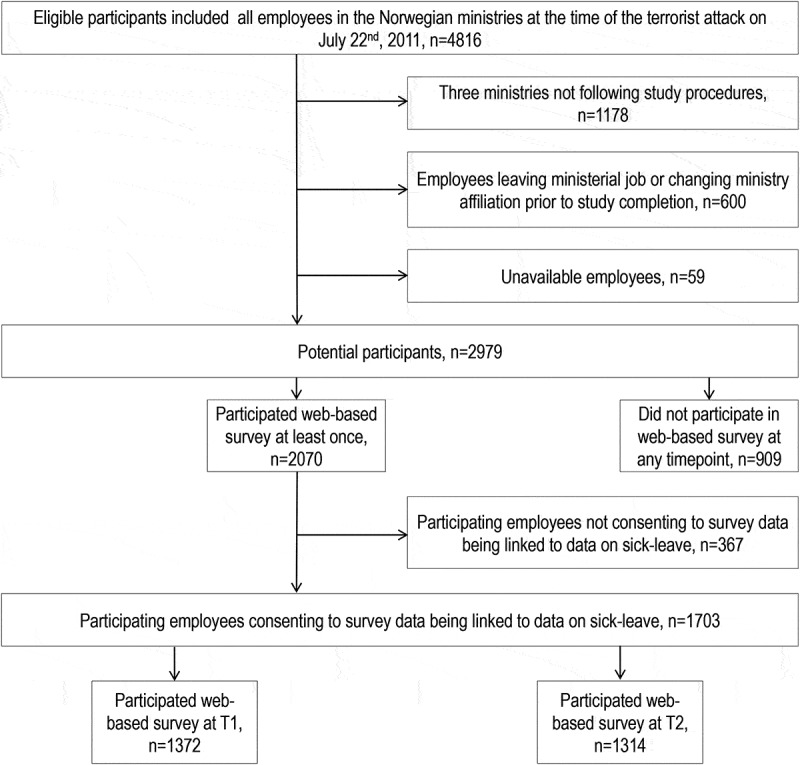
Flowchart of participants in the study on sick leave in association with perceived safety and threat among employees in the Norwegian ministries after the 2011 terrorist attack in downtown Olso, Norway.

The number of participants with missing data for key variable/models can be inferred from the tables. Because mixed effects models were used in analyses, participants contributed data to a model as long as they had at least one time point without missing values for model variables. T-tests and chi-square tests were used to evaluate selection bias at various stages in the study (see Figure 2). Point estimates are reported with 95% confidence intervals (CI) either in the tables or in the text. Demographic variables (age, gender and education) were included as covariates in the adjusted models because they were all significantly associated with either the predictors or the outcome, or both, in previous studies on the sample (Hansen, Berthelsen, Nissen, & Heir, 2019; Nissen et al., 2015, 2019). Exposure during the attack (‘present’ vs ‘not-present’) and PTSD were both included in adjusted models for the a priori reasons explained in the introduction. That is, an important aim of the study was to investigate whether perceived safety and threat could lead to sick-leave above and beyond that explained through the well-documented links between terrorism exposure, threat/safety perception and PTSD. Lastly, sick-leave prior to the attack was included as a potential confounder for theoretical reasons. First, sick-leave prior to the attack was expected to be strongly associated with sick-leave after the attack. And, second, sick-leave prior to the attack was hypothesized to be associated with personal characteristics (e.g. tendency to worry) that would also be associated with safety and threat perception.

The study’s primary aim was investigated with mixed effects hurdle models, with the count part modelled using a negative binomial distribution (Farewell, Long, Tom, Yiu, & Su, 2017; Rizopoulos, 2019; Zeileis, Kleiber, & Jackman, 2008). Hurdle models are suitable to analyse count data with an excess of zeros and overdispersion, both characteristics of sick-leave days in the present study. Hurdle models are two-part models. The first part uses logistic regression to estimate the odds of the outcome being above zero vs. zero for various predictor levels in the model, summarized as odds ratios (ORs) compared to a set reference. In the present study, the ORs compare the odds of having at least one day of sickness absence vs. no sickness absence for various predictor levels. The second part of the hurdle model uses truncated negative binomial regression for the data with a *positive* (i.e. above zero) outcome to estimate the count for various predictor levels in the model, summarized as a count or rate ratio (RR) compared to a set reference. In the present study, the RRs compare the number of sickness absence days for various predictor levels. To account for varying person-time at risk (i.e. not all employees worked full-time during the observation period), the hurdle models were offset by employees’ expected days of work in the relevant periods. Mixed effects, two-level, models were used since data was clustered within individuals given the study’s longitudinal design.

No pre-registered document with detailed analysis-plans exists, though the main aim of the study was developed before sick-leave data was obtained and can be found in prospectively written documents (e.g. grant proposal). The choice of covariates was largely determined by earlier work by the group on important predictors of perceived safety/threat in the present sample (Nissen et al., 2015, 2019; Nissen & Heir, 2016), and by study aims as described above. We have attempted to explain the rational behind how we handled variables and decided on models and analytic strategies.

Analyses were performed with Stata version 16 (STATA Corporation, College Station, TX, USA); and R, using the R package GLMMadaptive (Pinheiro & Bates, 1995; Rizopoulos, 2019)

## 3. Results

Figure 2 summarizes the flow of participants through the study. Of the 2979 potential participants prior to study start, 1372 (46.1%) answered the survey and gave consent that survey data could be linked to registry data on sick-leave at T1; and 1314 (44.1%) did so at T2. With reference to Figure 2, there was no statistical evidence that the 600 employees who quit their job or changed ministerial affiliation prior to study completion differed from the 1703 study participants on gender, age, education, perceived safety, trauma exposure or sick-leave, though strong evidence that the group had lower perceived threat (data not available for all subjects on all variables). There were proportionally more women (56.4 vs. 44.3%) in the group that participated at least once in the survey (n = 2070) compared to the group that never participated (n = 909), but no difference in age. When comparing the group that consented to survey data being linked to sick-leave data (n = 1703) to the group that did not (n = 367), there was some evidence that the consenting group was older (46.4 vs. 45.1 years) and strong evidence that it had more employees with > 16 years of education (64.3 vs. 52.4) and more people who felt safe at work. There was no evidence that the groups differed in terms of gender, perceived threat, trauma exposure or PCL mean item score.

Demographics and descriptive information are presented in Table 1. When comparing sick-leave in the years *after* the attack to sick-leave in the year *prior to* the attack using mixed effects hurdle modelling (not shown in tables), there was some evidence (*p* = 0.016) that the odds of sick-leave increased for the first year after the attack (OR = 1.24 [1.04–1.48]) and strong evidence (0.001 ≲ *p* < 0.002) that the length of sick-leave for those with sick-leave increased for all three years after the attack (RRs were 1.27 [1.12–1.45]; 1.23 [1.07–1.40]; and 1.35 [1.18–1.54] for year one, two and three after the attack, respectively). Since the first survey (T1) was done roughly one year after the attack, sick-leave period 1 corresponds to year 2 after the attack and sick-leave period 2 corresponds to year 3 (see Figure 1). Mean PCL item-scores for participants were 1.44 and 1.34 at T1 and T2, respectively. Symptom-based PTSD prevalences (PCL score ≥ 44) were 5.6 and 4.8 percent at T1 and T2, respectively.

**Table 1.** Descriptive statistics and distribution of variables for participating Norwegian ministerial employees after the 22 July 2011 terrorist attack in Oslo, Norway.

	T1 n = 1,372			T2 n = 1,314		
**Age, mean (SD)**	46.55	(10.56)		47.68	(10.52)	
Total	1,372			1,314		
**Sex, n (%)**						
Male	596	(43.4)		560	(42.6)	
Female	776	(56.6)		754	(57.4)	
Total	1,372	(100.0)		1,314	(100.0)	
**Education, n (%)**						
<13 years	150	(10.9)		165	(12.6)	
13–16 years	313	(22.8)		291	(22.2)	
>16 years	909	(66.3)		856	(65.2)	
Total	1,372	(100.0)		1,312	(100.0)	
**Feel safe at work**						
Mean (SD)	4.10	(1.09)		4.05	(1.05)	
Distribution, n (%)						
1 = Disagree	54	(4.0)		39	(3.0)	
2	84	(6.1)		88	(6.7)	
3	170	(12.4)		194	(14.8)	
4	415	(30.4)		431	(33.0)	
5 = Agree	643	(47.1)		556	(42.5)	
Total	1,366	(100.0)		1,308	(100.0)	
**Fear new attack at work, n (%)**						
Mean (SD)	1.73	(0.97)		1.96	(1.06)	
Distribution, n (%)						
1 = Disagree	726	(53.1)		546	(41.7)	
2	394	(28.8)		433	(33.1)	
3	160	(11.7)		203	(15.5)	
4	58	(4.3)		83	(6.4)	
5 = Agree	28	(2.1)		43	(3.3)	
Total	1,366	(100.0)		1,308	(100.0)	
**Sick-leave, n (%)**	Period 1*			Period 2*		
No	1,372	(74.6)		990	(75.3)	
Yes	349	(25.4)		324	(24.7)	
Length of sick-leave (days) for group with sick-leave, mean (min-max)	24.8	(1–190)		20.8	(1–186)	
Total	1,372	(100.0)		1,314	(100.0)	

* Period 1 and 2 equal the nine-month periods following the survey questionnaires at T1 and T2, respectively (see Figure 1).

Prior to adjusting for symptom-based PTSD, there was very strong evidence (all p-values except one < 0.001) that higher perceived safety was associated with both decreased odds of sick-leave and fewer sickness absence days for those with sick-leave (Model 1 and 2, Table 2). After controlling for PTSD, there was some evidence (0.021 < *p* < 0.034) that perceived safety was associated with reduced odds of sick-leave (logistic part of model 3), and moderate to strong evidence (0.004 < *p* < 0.013) that higher perceived safety was associated with fewer sickness absence days for those with sick-leave (count part of model 3). Both the reduction in the odds of sick-leave and the reduction in the number of sickness absence days were close to 30% in the fully adjusted model (upper 95% CI limits between 0.89–0.97).

**Table 2.** Two-part hurdle mixed effects models of sick-leave (Y/N) and weighted days of sickness absence regressed on perceived safety and threat among Norwegian ministerial employees after the 22 July terrorist attack in Oslo, Norway.

	Model 1		Model 2		Model 3	
	Logistic model, OR	Count model, RR	Logistic model, OR	Count model, RR	Logistic model, OR	Count model, RR
	(n = 1,621)	(n = 539)	(n = 1,617)	(n = 538)	(n = 1,617)	(n = 538)
**Perceived safety**						
Low	Ref.	Ref.	Ref.	Ref.	Ref.	Ref.
Medium	0.52 ^<0.001^	0.63 ^<0.001^	0.56 ^<0.001^	0.67 ^<0.001^	0.69 ^0.021^	0.70 ^0.004^
	[0.38 to 0.72]	[0.50 to 0.81]	[0.41 to 0.77]	[0.52 to 0.85]	[0.50 to 0.94]	[0.55 to 0.89]
High	0.46 ^<0.001^	0.64 ^<0.001^	0.52 ^<0.001^	0.70 ^0.003^	0.71 ^0.034^	0.73 ^0.013^
	[0.34 to 0.63]	[0.51 to 0.82]	[0.39 to 0.71]	[0.55 to 0.89]	[0.52 to 0.97]	[0.57 to 0.94]
	Model 4		Model 5		Model 6	
	Logistic model, OR	Count model, RR	Logistic model, OR	Count model, RR	Logistic model, OR	Count model, RR
	(n = 1,622)	(n = 539)	(n = 1,618)	(n = 538)	(n = 1,617)	(n = 537)
**Perceived threat**						
High	Ref.	Ref.	Ref.	Ref.	Ref.	Ref.
Medium	0.62 ^0.006^	0.67 ^0.002^	0.65 ^0.009^	0.71 ^0.007^	0.73 ^0.059^	0.74 ^0.019^
	[0.44 to 0.87]	[0.52 to 0.87]	[0.47 to 0.90]	[0.55 to 0.91]	[0.53 to 1.01]	[0.57 to 0.95]
Low	0.49 ^<0.001^	0.75 ^0.022^	0.53 ^0.001^	0.78 ^0.049^	0.62 ^0.002^	0.83 ^0.13^
	[0.36 to 0.68]	[0.59 to 0.96]	[0.39 to 0.72]	[0.62 to 1.00]	[0.45 to 0.84]	[0.65 to 1.06]

OR = odds ratio [95% CI] for sickness absence from work (Y/N). RR = rate ratio [95% CI] for length of sickness absence in those with sick leave. The n noted under each model indicates the number of unique individuals contributing data to a particular model. Data on employee perceived safety and threat were collected through two waves of survey questionnaires (roughly 10 and 22 months after the attack) and regressed on recorded sick-leave days for two nine-month periods following each survey. The three models adjusted for the following variables

Model 1: Time, gender, age, education.

Model 2: Model 1 variables + sick leave prior to attack + employee exposure (present versus not-present in the government quarter).

Model 3: Model 2 variables + symptom-based PTSD (from PCL sum-score).

The evidence for an association between perceived threat and sickness absence was weaker (Models 4–6, Table 2). Nonetheless, the models prior to adjusting for PTSD showed fairly strong evidence (0.001 < *p* < 0.009) that lower perceived threat was associated with reduced odds of sickness absence, and moderate evidence that lower perceived threat was associated with fewer sickness absence days in those with sick-leave (three of four *p*-values < 0.023). In the full models, however, the evidence of an association was not clear. There was strong evidence (*p* = 0.002) that the lowest level of perceived threat was associated with reduced odds of sick-leave compared to the reference group (high perceived threat). The reduction in odds was 38% (95%CI 16.0–55.0 percent). There was also some evidence (*p* = 0.019) that the second lowest level of perceived threat was associated with fewer sickness absence days (RR = 0.74, 95%CI 0.57–0.95). However, this was not the case for the lowest-level category of perceived threat. Overall, therefore, there was inconclusive evidence from the full models that perceived threat was associated with sick-leave.

## 4. Discussion

The present longitudinal study on more than 1700 employees in the Norwegian ministries whose workplace was subject to a terrorist attack showed clear evidence that higher perceived safety after the attack was associated with reduced odds of having sick-leave and fewer sickness absence days in those with sick-leave, even after adjusting for terror exposure and symptom-based PTSD. There was inconclusive evidence in the fully adjusted models that lower perceived threat was associated with reduced sick-leave. Feeling unsafe is common in terror-affected populations, even among the vast majority with no direct exposure to terrorism (Boscarino et al., 2006; Schuster et al., 2001; e.g. Silver et al., 2002). Thus, even if the true effects are close to the upper limit estimates of the present study, it might be of public health significance.

As far as we know, no other study has explored sick-leave in association with perceived safety/threat after workplace terrorism or other forms of violence. A prior study by our group has documented an overall increase in sick-leave in ministerial employees following the 2011 attack (Hansen et al., 2019), and several studies have found that exposure to workplace violence other than terrorism is linked to increased sick-leave (for and overview, see (Lanctôt & Guay, 2014)). These studies, however, did not look at the consequences of low perceived safety and elevated fears evident in a wide range of individuals after such events, including those with minimal or no direct exposure (e.g. Boscarino et al., 2006; Finseraas & Listhaug, 2013; Howie, 2007; Mainiero & Gibson, 2003; Marshall et al., 2007; North et al., 2015; Rubin et al., 2007; Schuster et al., 2001). Rather, the focus was on the immediate victims and the health consequences of direct exposure. An important aim of the present study was to explore whether safety/threat perception may result in sick-leave above and beyond that explained by the fairly established psychotraumatological links between trauma exposure, perceived safety/threat and PTSD (Ehlers & Clark, 2000; Fullerton et al., 2015; Grieger et al., 2003a, 2003b; Hansen & Elklit, 2011; Nissen et al., 2015). By providing evidence that low perceived safety is associated with increased sickness absence even after adjusting for terror exposure and symptom-based PTSD, the study adds empirical support to the importance of identifying and tailoring suitable interventions for the potentially high number of individuals with sub-diagnostic distress following terrorism that may suffer ill health even if they were not directly exposed and do not meet criteria for PTSD (North & Pfefferbaum, 2002). Devising and implementing strategies to make employees feel safe again after a workplace terrorist attack should be a priority for any employer for humane and ethical reasons. The present study indicates that these strategies may also positively impact health and reduce sick-leave. Future research may want to explore if certain strategies for making employees feel safe are more effective than others.

Findings are broadly in line with two prior studies by Holman et al. investigating fear and worry of terrorism and self-reported health conducted on large, population-based samples without direct exposure to terrorism (Holman et al., 2019, 2008; Melamed et al., 2004). One of these studies identified fear of terrorism as an important mediator of the positive association between second-hand media exposure to terrorism and functional impariment due to physical and emotional health issues six months later (Holman et al., 2019). The other found that ongoing worry about terrorism acted as an effect modifier in the assocation between acute stress reactions following terrorism and self-reported, physician-diagnosed, cardiovascular ailments (Holman et al., 2008). The present study modelled and tested perceived threat and safety as the direct predictors of ill health, and not as mediators or modifiers. This was partly based on Ehlers and Clark’s cognitive model of PTSD where perceived threat is though to be the key early feature of the disorder, later accompanied by the other PTSD symptoms (Ehlers & Clark, 2000). One could argue, though, that second-hand exposure through media and/or ‘second-hand’ exposure through returning to a workplace devastated by a massive explosion were important and necessary factors driving the associations between perceived safety and sick-leave in the present study. It could also be that acute stress reactions in the immediate aftermath of the attack played a role in explaining the associations. That is, acute stress reactions may have caused employees to start worrying about terrorism and workplace safety, which subsequently raised sick-leave rates. Establishing the best way to model variables and their correct causal order is not necessarily easy or straightforward. For example, acute stress reactions may partly overlap with perceived threat and safety, both conceptually and temporally.

One element separating the present study from the two studies by Holman et al. is that data on health in the present study was based on records from national databases on sick-leave and not self-reported health. This may have the advantage of reducing potential problems with common methods bias. On the other hand, self-reported health may be a more sensitive measure of health than doctor-certified sickness absence as a person could certainly suffer ill health and still manage to go to work – i.e. ill health and sickness absence represent different, though overlapping concepts (Wikman, Marklund, & Alexanderson, 2005). Melamed et al. also used non-self report data on health when exploring possible health consequences of terrorism fears (Melamed et al., 2004). This study found that fear of terrorism was associated with an elevation in the inflammatory marker, C-reactive protein (CRP), in adult, working women undertaking routine hospital screenings in Israel (the association was not found in men). CRP is thought to be a risk factor for cardiovascular disease and diabetes among other things.

It is worth noting that the populations and settings in the studies by Holman et al., the study by Melamed et al. and the present study were rather different. Study subjects in the Holman et al. studies came from representative national samples (few or none of which had been directly exposed to terrorism). Subjects in the Melamed et al. study were healthy adults living in or near Tel Aviv, Israel (presumably some or many of which had had some degree of exposure to terrorism given the regional conflicts). Subjects in the present study were employees in the Norwegian ministries, of which roughly 10% where in geographical proximity when the terrorist attack on the ministries occurred. Categorizing the other 90% as indirectly exposed appears overly simplistic as many employees saw their offices completely shattered, many had to move location due to damage to infrastructure and most knew someone that was seriously hurt or died in the attack. Therefore, a notably higher proportion than 10% likely met the A criterion for PTSD in DSM-V. Despite these differences in population and setting, and despite using different measures of health, all studies point to fear of terrorism as a potential contributor to ill health following terrorism.

Adjusting for symptom-based PTSD in the final models probably leads to an underestimation of the true association between perceived safety/threat and sick-leave in our study. That is, since PTSD is likely to mediate at least part of this association (Ehlers & Clark, 2000), the ORs and RRs in the final models, in theory, indicate the strength of the association other than that explained by PTSD. Therefore, models 2 and 5 in Table 2 reflect more accurately the true strength of association between perceived safety/threat and sick-leave. Whether interventions aimed at limiting distortions in threat and safety perception following workplace terrorism may indirectly impact the overall terror-related PTSD burden in workers should also be examined in future studies.

### 4. 1. Limitations and strengths

Even if predictors were measured prior to outcome, we still cannot make conclusions about causality. One concern with our primary conclusion is that there were very strong associations between perceived safety/threat *after* the attack and sick-leave *prior* to the attack, even after adjusting for potential confounders. This may indicate some underlying, intra-individual, characteristic or vulnerability predisposing to lower levels of perceived safety, elevated levels of perceived threat, as well as sick-leave. By controlling for sick-leave prior to the attack, we attempted to control for this potential underlying confounder, though we cannot exclude residual confounding or indeed confounding by other important factors not included in the study.

As summarized in the Results Section, there is limited statistical evidence to suspect major issues with selection bias. However, we cannot exclude that participation was associated with the primary outcome of the study (sick-leave), since the distribution of the surveys were mostly done at work. It is therefore possible that proportionally too few employees with sickness absence participated. Sick-leave may have been further underestimated because consent to using registry data was strongly positively associated with education which was strongly negatively associated with sick-leave. On the other hand, it is possible that employees with little or no sick-leave found the study less relevant and interesting and decided not to participate, possibly leading to an overestimation of sick-leave in the study.

Another weakness was that predictors were measured through single-item statements with limited psychometric data available, and the question on perceived safety had no reference to terrorism in the statement (i.e. may have tapped feelings of unsafety unrelated to terrorism). Sick-leave was confined to *doctor-certified* sick-leave. Sick-leave rules in Norway are quite liberal and flexible, and most workers are allowed to be home from work due to illness for up to three consecutive days, four times per year, without a sick-leave note from a doctor. It is likely, therefore, that the overall *absenteeism* from work due to health reasons was even higher than the study’s estimate, which relied solely on doctor-certified sick-leave.

Lastly, without a detailed and predetermined analysis plan, we cannot exclude that analysis has been influenced by the data.

## 5. Conclusions and recommendations

The key message from the present study was that low perceived safety in employees after a workplace terrorist attack was positively associated with subsequent sick-leave, and that this sick-leave may not be sufficiently accounted for by focusing on trauma exposure and PTSD. The study gives empirical support to the argument that post-disaster response plans and interventions should include strategies on how to identify and approach the potentially large number of individuals who may suffer adverse health consequences after terrorism even if they have limited direct exposure and do not meet criteria for PTSD (e.g. Marshall et al., 2007; North et al., 2011, 2015; North & Pfefferbaum, 2002). It further supports the potential value of effective risk communication as an important intervention after traumatic events which often lead to distortions in risk appraisals and excessive fear (Marshall et al., 2007; North et al., 2010). Specific strategies employers may follow in the aftermath of workplace terrorism include distributing information and educating employees on escape- and evacuation procedures, and communicating and showing a clear commitment to safety and security measures (Nissen et al., 2019; Nissen & Heir, 2016).

## Data

Selected data will be made available by contacting the first author, respecting agreements made with the ethical board and participants.

We declare no conflict of interest with regards to our recently accepted manuscript ‘Is perceived safety and threat after workplace terrorism linked to employee sick-leave? A registry-based longitudinal study of governmental employees in Norway’, Mr Alexander Nissen, *European Journal of Psychotraumatology* (Ms. No. ZEPT-2019-0177R3).
